# Histamine H_2_-receptor antagonism improves conduit artery endothelial function and reduces plasma aldosterone level without lowering arterial blood pressure in angiotensin II–hypertensive mice

**DOI:** 10.1007/s00424-024-02909-0

**Published:** 2024-01-27

**Authors:** Kasper B. Assersen, Boye L. Jensen, Camilla Enggaard, Paul M. Vanhoutte, Pernille B. L. Hansen

**Affiliations:** 1 Cardiovascular and Renal Research, University of Southern Denmark, J. B. Winsløwsvej 21, Odense C, DK-5000 Odense, Denmark; 2 State Key Laboratory of Pharmaceutical Biotechnology and Department of Pharmacology and Pharmacy, University of Hong Kong, Hong Kong, China

**Keywords:** Hypertension, Aorta, Mesenteric artery, Myography

## Abstract

Aldosterone through the mineralocorticoid receptor MR has detrimental effects on cardiovascular disease. It reduces the bioavailability of nitric oxide and impairs endothelium-dependent vasodilatation. In resistance arteries, aldosterone impairs the sensitivity of vascular smooth muscle cells to nitric oxide by promoting the local secretion of histamine which activates H_2_ receptors. The present experiments tested *in vivo* and *ex vivo* the hypothesis that systemic H_2_-receptor antagonism reduces arterial blood pressure and improves vasodilatation in angiotensin II–induced chronic hypertension. Hypertension was induced by intravenous infusion of angiotensin II (60 ng kg^−1^ min^−1^) in conscious, unrestrained mice infused concomitantly with the H_2_-receptor antagonist ranitidine (27.8 µg kg^−1^ min^−1^) or vehicle for 24 days. Heart rate and arterial blood pressure were recorded by indwelling arterial catheter. Resistance (mesenteric) and conductance (aortae) arteries were harvested for perfusion myography and isometric tension recordings by wire myography, respectively. Plasma was analyzed for aldosterone concentration. ANGII infusion resulted in elevated arterial blood pressure and while *in vivo* treatment with ranitidine reduced plasma aldosterone concentration, it did not reduce blood pressure. Ranitidine improved *ex vivo* endothelial function (acetylcholine 10^−9^ to 10^−6^ mol L^−1^) in mesenteric resistance arteries. This was abolished by *ex vivo* treatment with aldosterone (10^−9^ mol L^−1^, 1 h). In aortic segments, *in vivo* ranitidine treatment impaired relaxation. Activation of histamine H_2_ receptors promotes aldosterone secretion, does not affect arterial blood pressure, and protects endothelial function in conduit arteries but promotes endothelial dysfunction in resistance arteries during angiotensin II–mediated hypertension. Aldosterone contributes little to angiotensin II–induced hypertension in mice.

## Introduction

Activation of mineralocorticoid receptors (MRs) by aldosterone can contribute to the elevation of arterial blood pressure [6, 17, 47, 48] and impaired vasodilatation (i.e., vascular dysfunction) [17, 47, 60] in hypertension. High arterial blood pressure, vascular dysfunction [38, 40], and augmented serum levels of aldosterone [27] independently predict cardiovascular events and mortality. Insufficient blood pressure control by inhibition of angiotensin-converting enzyme (ACE) or angiotensin II receptors is experienced when circulating levels of aldosterone are increased [47], possibly due to aldosterone “breakthrough” (serum aldosterone levels returning to normal following initial depression during ACE inhibition or angiotensin II receptor blockade). In humans, the aldosterone-dependent blood pressure elevations and impaired vasodilatations are, at least in part, due to extra-renal effects [17, 39]. In mice, direct vascular effects of the hormone have been demonstrated. Thus, arterial blood pressure increased by inducible overexpression of MR in endothelial cells [45] whereas conditional MR knock-out in vascular smooth muscle cells reduced vascular contractility and improved endothelial function in age-related and angiotensin II–induced hypertension [42]. Pro-contractile effects of aldosterone in resistance arteries could explain these MR-dependent effects on blood pressure. In isolated rodent mesenteric arteries, endothelial dysfunction was elicited *in vitro* by the administration of exogenous aldosterone [43, 51]. These direct vascular effects of aldosterone, at least in mesenteric arteries of wild-type mice, were attributable to the local release of histamine [10, 51] and the subsequent activation of histamine type 2 (H_2_) receptors which reduced the sensitivity of the vascular smooth muscle cells to nitric oxide (NO) [51]. In addition, during H_2_-receptor inhibition, the concentration-contraction curve to cumulative concentrations of histamine was shifted to the right in human mammary and radial artery segments, indicating pro-contractile properties of H_2_ receptors [11]. Hence, aldosterone may promote the exaggerated release of histamine either by mast [51] or other cells in the vascular wall [10], with subsequent activation of H_2_ receptors to promote a pro-contractile state in resistance arteries and increased arterial blood pressure. This may contribute to the harmful MR-dependent cardiovascular effects during increased circulating levels of aldosterone. However, most observations suggesting such involvement of histamine were obtained in vascular preparations studied *ex vivo*. Therefore, the present experiments were designed to test the hypothesis that histamine H_2_-receptor antagonism reduces arterial blood pressure *in vivo* and improves endothelial function, as studied *ex vivo*, in angiotensin II–induced hypertension in the mouse. To address this hypothesis, conscious unrestrained mice, instrumented with chronic arterial indwelling catheters, were studied during continuous angiotensin II infusion, without or with concomitant H_2_ receptor blockade. At the end of the *in vivo* infusions, *ex vivo* experiments were conducted in isolated mesenteric arteries and aortae by isobaric perfusion myography and isometric tension recordings, respectively.

## Materials and methods

### Animals

The experimental protocols were approved by the Danish National Animal Experiments Inspectorate under the Danish Ministry of Environment and Food (2015-15-0201-00479). Animal care was carried out in accordance with the Animal Experiment Law. Studies were conducted on C57BL/6NTac (Taconic Farms Inc., Ejby, Denmark). The mice were acclimatized for at least 6 days before undergoing surgery. They were housed in The Biomedical Laboratory’s Animal Housing Facilities, in a controlled environment (12:12 h light/dark cycle, 21 ± 3 °C, 55 ± 15% humidity) and had free access to water and standard chow (#1324, Altromin, Brogaarden, Hørsholm, Denmark).

### *In vivo* experiments: continuous arterial blood pressure and heart rate measurements

**Fig. 1:**
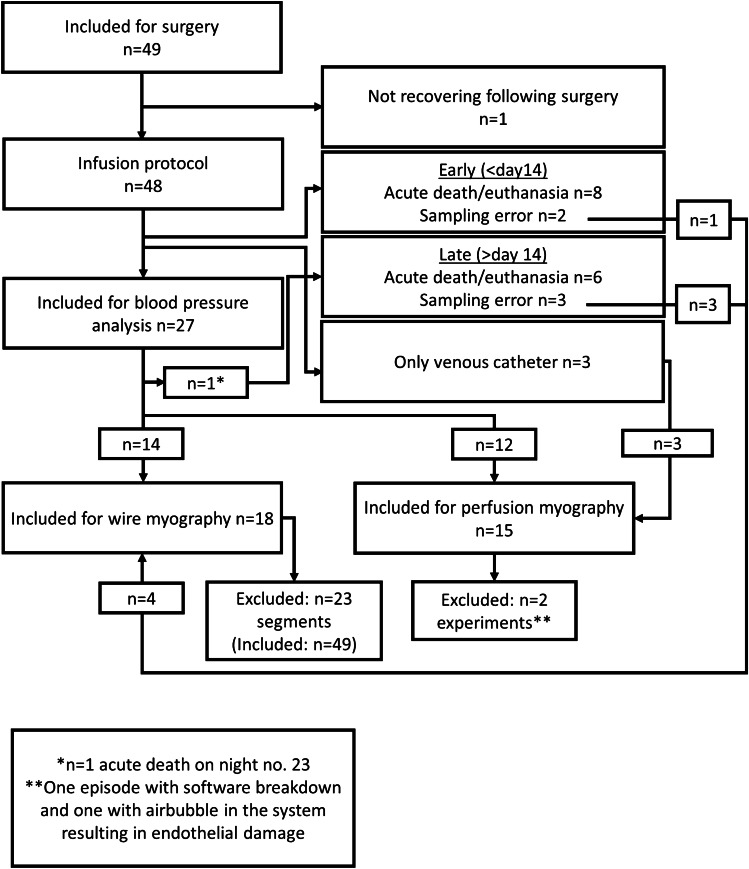
The flowchart displays the inclusion and distribution of animals in the experimental series. Twenty-seven animals were included for blood pressure analysis, 18 for wire myography, and 15 for perfusion myography. The mice that completed the infusion protocol were included in myography (either wire or perfusion). Therefore, animals which were not included in the blood pressure analysis due to technical sampling error (i.e., data not recorded by the software) and animals who were only implanted with a venous catheter could still be included for wire/perfusion myography

Before surgery, mice were allocated either to continuous infusion of angiotensin II (AngII, diluted in heparinized isotonic glucose solution (100 IU mL^−1^), infusion rate 60 ng kg^−1^ min^−1^) [36] to induce hypertension or to infusion with the histamine H_2_-receptor antagonist ranitidine (27.8 µg kg^−1^ min^−1^) [24] in combination with AngII (ranitidine+AngII). During anesthesia (100 mg kg^−1^ ketamine and 10 mg kg^−1^ xylazine I.P.), chronically indwelling catheters were surgically implanted into the femoral artery and vein of mice (aged 7-12 (AngII 9.0 ± 0.2 ranitidine+AngII 9.3 ±0.2) weeks) as described [2, 21, 56, 57]. After 3 days of recovery, ranitidine infusion was initiated at 27.8 µg kg^−1^ min^−1^ (10 µl h^−1^, diluted in heparinized glucose). After 5 days of recovery, data collection of baseline intra-arterial blood pressure and heart rate was initiated by connecting the intraarterial catheter to a pressure transducer (Föhr Medical Instruments, Hessen, Germany). Data were recorded at 100 Hz with LabView (National Instruments, Austin, TX, USA). After 4 hours of baseline blood pressure and heart rate recording, AngII was added to the heparinized glucose, and infusion was initiated at 60 ng kg^−1^ min^−1^ (10 µl h^−1^ with and without simultaneous ranitidine infusion (ranitidine+AngII)). Infusions continued for 24 days. Fifty-three mice underwent surgery of which 34 animals were included for data analysis (Fig. 1). Animals were excluded when not recovering following surgery (*n*=1), due to early (<14 days) or late acute death (*n*=2 and *n*=5), euthanasia (early *n*=6 and late *n*=1) or sampling errors (early *n*=2 and late *n*=3). The sampling errors leading to exclusion were broken/malfunctioning arterial catheters (*n*=4, i.e. IV infusion intact) and no data sampled during baseline (*n*=1). For the latter there was uncertainty whether the infusion had been compromised for the duration of time where no data was sampled, and therefore the animal was fully excluded.

### Sampling and tissue isolation

After 24 days of treatment, blood samples from the arterial catheter (2×200 µL) were collected in microcentrifuge tubes on ice, followed by centrifugation (3000 RPM, 10 min, 5 °C), plasma isolation, and snap-freezing followed by storage at −80 °C until analysis. Immediately after blood sampling, the animals were killed by stunning by a blow to the head, followed by decapitation. Animals were weighed, and their thoracic aorta and intestines were harvested. Hearts were also isolated, weighed, stored in a microcentrifuge tube, and snap-frozen in liquid nitrogen for storage at −80 °C.

### Isolation and microperfusion of mesenteric arteries

Second-order mesenteric arteries were cleaned of perivascular adipose tissue, isolated in 4 °C control medium (see composition below) and studied in a perfusion setup, as described [51]. Briefly, the preparations were mounted with nylon sutures on glass pipettes in an organ chamber (in-house custom made, stainless steel chamber with glass floor, 2 mL volume). Arteries were perfused with a control medium (supplemented with FBS (1%)) applying a pressure gradient of Δ5 mmHg (60 mmHg at the inlet) resulting in a constant perfusion with a 1–2 µl min^−1^ flow rate [51]. The chamber contained a control medium, supplemented with fetal bovine serum (FBS, 0.1%), which was exchanged at the rate of 16.5 mL h^−1^; the temperature of the chamber was maintained at 37 °C by a thermostat controller (TC-324B temperature controller, Warner Instruments, Hamden, CT, USA). The control medium was perfused with 5% CO_2_ in the air and was not applied to the setup until equilibrium after pH had been adjusted by the addition of HCl or NaOH to pH=7.4 ± 0.02. The pH was stabilized by an ambient local atmosphere of heated 5% CO_2_ in the air. The chamber rested on an inverted microscope (Zeiss Axiovert 10, Oberkochen, Germany) connected to a camera. Software (Till Photonics, Munich, Germany) allowed during interventions for live-recording of vasomotion with a personal computer. After mounting, perfused arteries were allowed to develop a spontaneous tone for 30 min before experiments were conducted. To test viability, arteries were first stimulated with an extraluminal addition of 100 mmol L^−1^ KCl. Following 30 min of rest, they were constricted with U46619 (10^−7^ mol L^−1^; thromboxane A2 receptor (TP) agonist [5]) and dilated with the endothelium-dependent vasodilator acetylcholine ([10^−5^ mol L^−1^; muscarinic agonist [18]]. Since *in vivo* angiotensin II treatment was expected to induce endothelial dysfunction [20], no fixed inclusion cutoff was defined for dilatation. Arteries were included if they responded with dilatation to 10^−5^ mol L^−1^ acetylcholine during the viability test which averaged 69% ± 4 for ranitidine+angiotensin II–treated animals and 64% ± 5 for angiotensin II–treated animals.

The experimental series strictly adhered to a pre-defined time schedule, giving arteries 3 min to respond to each concentration of the compound. Before and after each experimental series, arteries were allowed to rest for 30 min. Following the viability test, arteries were constricted by extra-luminal addition of cumulative concentrations of U46619 (10^−9^, 10^−8^, 3×10^−8^, 6×10^−8^, 10^−7,^ and 10^−6^ mol L^−1^), after which they were washed with FBS supplemented control medium. Dilatation was tested with the extra-luminal addition of cumulative concentrations of acetylcholine (10^−9^, 10^−8^, 10^−7^, and 10^−6^ mol L^−1^), in arteries which were submaximally pre-constricted with U46619 (10^−7^ mol L^−1^). For experimental protocol, see Fig. 4.

### Isometric tension recordings

The thoracic aortae were submerged in a 4 °C control medium (composition below) and cleaned of periaortic fat. The aortic arch was removed, and the remaining aorta was divided to yield four segments (approximately 2 mm in length) which were, by random allocation, suspended between stainless steel pins (200 µm diameter) in the chambers of a four-chamber wire myograph (Multi Wire Myograph 610m, DMT, Aarhus, Denmark). Isometric tension was recorded, with an analogue-to-digital signal-converter (PowerLab 4/30 & Maclab 8e; ADInstruments, Sydney, Australia) connected to a computer which recorded data by the software Labchart (ADInstruments). Chambers contained 5 ml of pH-adjusted control medium kept at 37 °C and aerated with 5% CO_2_ in the air to maintain pH at 7.4 throughout the experiment. The equipment was calibrated, and the arterial segments were normalized in accordance with the manufacturer’s instructions to a passive tension corresponding to a distending pressure of 100 mmHg. Following normalization and between each experimental sequence, segments were allowed to equilibrate for at least 30 min. The viability of the vascular smooth muscle cells was evaluated by two consecutive stimulations with a high extracellular concentration of potassium solution (100 mmol L^−1^ KCl) in the presence of the α-adrenergic antagonist phentolamine (10^−5^ mol L^−1^, 5 min to block possible effects of endogenous noradrenaline) [35]. Segments were included for data analysis if tension development to 100 mmol L^−1^ extracellular K^+^ exceeded 1 N m^−1^. The viability of the endothelial cells was determined by pre-contracting the segments with U46619 (10^−8^ mol L^−1^) [5] and subsequently relaxing them with acetylcholine (10^−5^ mol L^−1^) [51]. As for mesenteric arteries, no fixed inclusion cutoff was defined for relaxation which was 55% ± 4 for ranitidine+angiotensin II and 49% ± 3 for angiotensin II–treated animals. During the recording of the responses to cumulatively increasing concentrations of agonist, the concentration was increased every 3 min for acetylcholine (ACh) and for diethylamine NONOate (10^−9^ to 10^−5^ mol L^−1^; DEA NONOate; NO donor). For U46619 cumulative concentration-contraction measurement, concentrations were increased when maximal force had developed. Each of the four experimental conditions were randomly assigned to individual segments (Fig. 5D): (a) time control, (b) untreated segment (≠ time control—designated for aldosterone treatment—see below), (c) +eplerenone (10^−5^ mol L^−1^, 30 min pre-incubation) [4], and (d) +ranitidine (10^−5^ mol L^−1^, 30 min pre-incubation) [30]. For all segments, the concentration-contraction curve to U46619 was established initially. After 30 min of equilibration, relaxations to cumulative concentrations of acetylcholine were obtained in segments pre-contracted with U46619 (EC_50_). The effect of incubation with aldosterone (10^−9^ mol L^−1^, 1 h [51]) on relaxations to acetylcholine was then determined in all segments (excluding the time control). Finally, relaxations to cumulative concentrations of diethylamino NONOate were obtained (see the experimental protocol in Figure 5D). During the initial half of the wire-myograph experiment, the arterial segments which are designated for time control and those designated for later aldosterone treatment are treated the same way (see Figure 5D). This was chosen to allow for parallel comparisons of time control, aldosterone, and eplerenone+aldo/ranitidine+aldo-treated groups without introducing any time bias (if aldosterone treatment was initiated for the aldo-groups at baseline, we would have introduced a potential treatment duration bias during parallel comparisons). Segments were selected for a condition by drawing lots at random before performing the viability test. As some arterial segments were not viable during the viability test and these segments had already been randomly allocated for their treatment condition, five viable segments from angiotensin II–treated mice ended up with time-control conditions, and seven viable segments from angiotensin II–treated mice ended up with (later) aldosterone conditions. As to not exclude data from viable segments but also not include more than one segment per animal under the same conditions, results from segments under time-control conditions (that is, both designated time-control segments and designated (later) aldosterone segments) were included as *n*=1. This led to *n*=7 time-control segments in Fig. 5A, consisting of two designated (later) aldosterone segments under time-control conditions and ((5+5)/2) paired time-control and (later) aldosterone segments under time-control conditions. In Figure 6A, only the designated (*n*=5) time-control segments provide data to the graph, whereas the *n*=7 segments designated for aldosterone treatment are included in Fig. 6B, as they had then been treated with aldosterone.

### Plasma aldosterone analysis

Plasma aldosterone (determined in two paired blood samples, taken from the femoral artery catheter, in immediate succession on day 24 and not preceded by other blood samples) was measured using a commercially available kit (LDN, Nordhorn, Germany, Cat. No. MS E-5200). Briefly, an antibody-coated 96-well plate was loaded with standards, control samples, mouse plasma, and an aldosterone-horse radish peroxidase conjugate facilitating competitive binding. The aldosterone concentration is proportional to the signal intensity of an enzymatically catalyzed colorimetric reaction as detected by spectophotometry at 450 nm wavelength. Human EDTA–treated plasma was used as an internal standard. The mean arterial plasma aldosterone concentration was 70.4 ± 9.6 pg ml^−1^, and the interassay variation from a human plasma pool sample was 8.6%.

### Chemicals, compounds, and solutions

For anesthesia, ketamine (50 mg mL^−1^, MSD animal health, Kenilworth, NJ, USA) and xylazine (Rompun vet, 20 mg mL^−1^, Bayer Animal Health GmbH, Leverkusen, Germany) were injected intraperitoneally. For infusions, ranitidine (ranitidine hydrochloride, Sigma-Aldrich, St. Louis, MO, USA) was dissolved in isotonic glucose (55 mg mL^−1^) to a stock concentration of 12 mg ml^−1^ stored at −20 °C. Angiotensin II (Sigma-Aldrich) was dissolved in acetic acid (100%) to 20 mg mL^−1^, further diluted in isotonic glucose to a stock concentration of 0.2 mg mL^−1^, and stored at −20 °C. All dilutions were performed in heparinized glucose (100 IU mL^−1^). Control medium [21, 36] consisted of (in mol L^−1^) NaCl 115, NaHCO3 25, MgSO4 1.2, K2HPO4 2.5, CaCl2 1.3, glucose 5.5, and 4-(2-hydroxyethyl)-1-piperazineethanesulfonic acid (HEPES) 10 (Sigma-Aldrich for all compounds). High-concentration potassium solution [36] consisted of (in mol L^−1^) KCl 95, NaCl 20, NaHCO3 25, MgSO4 1.2, K2HPO4 2.5, CaCl2 1.3, glucose 5.5, and HEPES 10 (Sigma-Aldrich for all compounds). For *in vitro* experiments ranitidine, acetylcholine (Sigma-Aldrich) and phentolamine (phentolamine hydrochloride, Sigma-Aldrich) were dissolved to 10^−2^ mol L^−1^ in ultra-purified water before each experiment. U46619 (Tocris, Bio-Techne Ltd., Abingdon, UK) was dissolved in methyl acetate by the manufacturer and further diluted in ultra-purified water before each experiment. Aldosterone (Sigma-Aldrich) was dissolved in ethanol to a stock solution of 10^−2^ mol L^−1^, stored at −20 °C, and diluted in ultra-purified water to 10^−6^ mol L^−1^ before each experiment. Due to its short half-life (2 min at 37 °C [41]), diethylamine NONOate was dissolved to 10^−1^ mol L^−1^ in ultra-purified water and stored at −80 °C and was thawed and diluted in ice-cold ultra-purified water and kept on ice when its addition was imminent. Eplerenone (Sigma-Aldrich) was dissolved in dimethylsulfoxide (DMSO) to 10^−2^ mol L^−1^ and kept at −20 °C until shortly before addition.

### Data handling and statistical analysis

#### Arterial blood pressure and heart rate

Data, collected at 100 Hz, were averaged for 12-h periods (day/night) and applied for generation of curves. To exclude abnormal values (i.e., clear outliers, physiologically improbable values), assessed to be of a technical/mechanical nature, cutoff limits for blood pressure and heart rate (HR) were defined *post hoc*. As such, a 12-h average (night/daytime average) of mean arterial blood pressures (MAPs) was excluded if it was below the lowest average of the 4-h MAP recorded during baseline (91 and 93 mmHg for AngII and ranitidine+AngII mice, respectively). Cutoff values for heart rates were defined as the lowest 12-h average during acute angiotensin II–induced heart rate depression (cutoff 462 bpm for AngII and 456 bpm for ranitidine+AngII). All measures of MAP and HR below these thresholds in otherwise healthy, thriving animals, were interpreted as technical interruptions. In total, missing and excluded values did not exceed 7% of any dataset.

The effects on blood pressure and heart rate of the infusions were assessed by one-way analysis of variance (ANOVA). The effects of ranitidine and angiotensin II on blood pressure and heart rate were compared by calculating areas under the curve (AUC) which were compared by *Student’s t*-test for general effects (i.e., curve vs curve) and *Holm-Sidák’s* multiple comparisons test for specific effects (i.e., MAP average vs MAP average) using GraphPad Prism (GraphPad Software Inc., San Diego, CA, USA).

#### Plasma aldosterone comparison

Plasma aldosterone was compared by *Student’s t-*test.

#### Isolated perfused mesenteric arteries

Video recordings of vascular experiments were exported from the recording software and analyzed using the imaging software ImageJ [52]. From the imaging software, luminal diameter was manually measured and extracted for every 5 s of recording. The resting diameter after the development of spontaneous myogenic tone was defined as 100% relaxation. Diameter changes are displayed as changes from the precontracted diameter, which was defined as 0% relaxation. Diameters in absolute values (µm) were generated by normalizing extracted diameters to the length value of a snapshot of a 1-mm scale (Olympus, Hamburg, Germany). Concentration-response curves for normally distributed data were fitted with a non-linear regression curve, which had a fixed upper limit (maximal relaxation) of 100% dilatation, as no plateau was reached. As such, EC_50_ values were extracted from each curve and were compared by *Students t*-test for EC_50_. To compare obtained maximal responses, *Holm-Sidák’s* multiple comparisons test was applied to the data sets.

#### Heart weight

Heart weights were compared by *Student’s t-*test.

#### Isometric tension recordings data processing

Data extraction was performed using Labchart 8 Reader (ADInstruments). For data extracted to generate concentration-response-curves to U46619, acetylcholine, and DEA NONOate, a mean value of the tension curve for each concentration of agonist (i.e., a mean value of all data points from each concentration interval on the curve from between 10^−9^ and 10^−8^ mol L^−1^, between 10^−8^ and 10^−7^ mol L^−1^) was retained, to avoid selection bias from oscillating data and assure comparability. Extracted data is normalized to the active tension induced by extracellular [K^+^] of 100 mM for U46619-induced contractions and to the respective baseline tension (defined as 100%) and precontracted tension (0%) for relaxations. Data in absolute values are in mN mm^−1^. Concentration-response curves for normally distributed data were fitted with a non-linear regression curve and EC_50_ and E_max_ were compared by *F*-test. Before aldosterone incubation, segments (a) and (b) (see section “Isometric tension recordings” for definitions) were tested under the same experimental conditions. Therefore, results from these segments were averaged and included as *n*=1 for all data prior to aldosterone incubation.

#### General statistics

Data was assessed for normality by frequency distribution and quantum-quantum plotting. Data are shown as means ± SEM; *n* represents the number of individual animals contributing to each group, unless otherwise specified. One-way ANOVA was applied when comparing baseline diameters and viability tests of more than two groups (paired within, unpaired between) and Student’s *t-*test for groups of two. All analyses were performed using GraphPad Prism 6.07 (GraphPad Software Inc.).

## Results

A CONSORT-like flowchart shows the inclusion of mice, loss of mice during experimental protocols, and the number of mice at the completion of all experiments in Fig. 1. Moreover, the numbers of vascular rings harvested for the ex vivo experimental series from the in vivo series are shown in Figure 1. There was no systematic bias in mouse survival relating to the infusion of H_2_ receptor antagonists, and thus, vascular rings were equally distributed among groups.

### Effect of ranitidine on AngII-induced hypertension—*in vivo* measurements

#### Mean arterial blood pressure

**Fig. 2:**
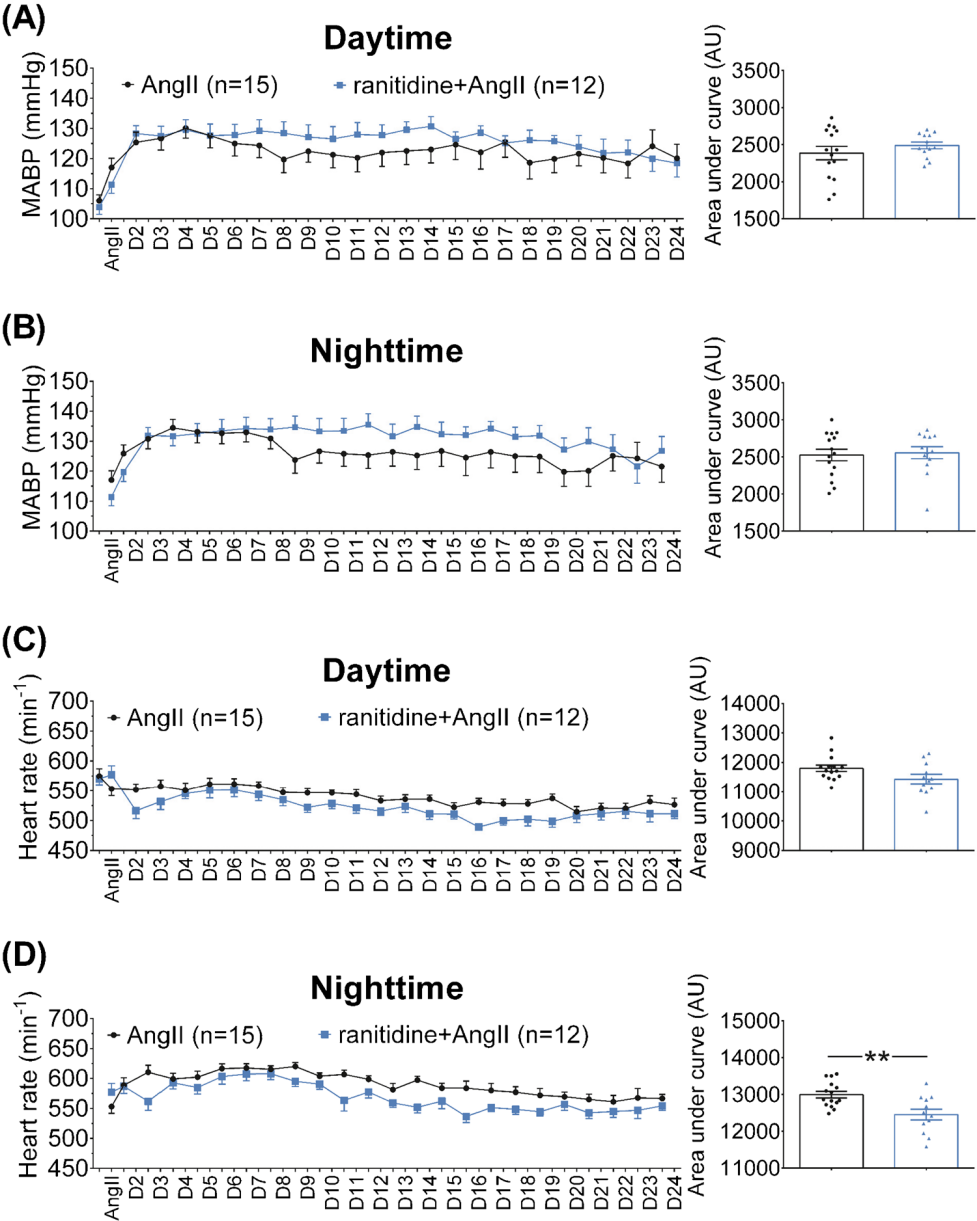
The panels show recordings of mean arterial blood pressure (**A** and **B**) for 24 days (D1–D24) and heart rate (**C** and **D**) responses to AngII infusion as measured via indwelling femoral artery catheter at 100 Hz in 15 i.v. angiotensin II+vehicle (AngII) and 12 ranitidine+angiotensin II (ranitidine/R+AngII)-treated C57BL/6 mice. Blood pressure measurements were averaged for every 12 h. Daytime blood pressures are labeled with D1–D24 (**A**), whereas nighttime is indicated by ticks on the *x*-axis in **B**. Blood pressure was not different in the two series and increased similarly and significantly in AngII (*P*<0.0001, ANOVA) and ranitidine+AngII- (*P*<0.0001, ANOVA) treated animals (**A** and **B**). Integrated long-term blood pressure responses were statistically similar as indicated by no difference in area under the curve (AUC) in diagrams shown on the right side ((**A**) daytime *P*=0.3, **B** nighttime *P*=0.8, *Student’s t-*test) after maximal blood pressure response. Heart rate transiently decreased following AngII-infusion in ranitidine+AngII-treated animals (**A**) *P*=0.002, *Holm-Sidák’s multiple comparisons test*) but not in AngII-treated animals (**A**) *P*=0.2, *Holm-Sidák’s multiple comparisons test*). Heart rate responses were statistically similar during daytime (**C**), but differed significantly between treatment groups during nighttime, as the area under the curve was significantly different ((D) *P*<0.01, *Student’s t-*test). The normality of residuals was evaluated by frequency distribution and quantum-quantum plot. Data are shown as means ± SEM

Baseline MAP was stable and statistically similar in the two series before treatment (AngII 108 ± 2 mmHg and ranitidine+AngII mice 104 ± 2 mmHg, *P*>0.99, Fig. 2A, B). AngII infusion significantly augmented MAP (maximum 134 ± 3 mmHg, *P*<0.0001), and ranitidine did not alter the hypertensive response (maximum 134 ± 3 mmHg, *P*<0.0001) to angiotensin II infusion as tested with Holm-Sidák multiple comparisons test. Also, no difference in AUCs was detected (daytime 2387 ± 90 vs 2490 ± 46 AU, *P*=0.34, nighttime 2525 ± 79 vs 2555 ± 80 AU, *P*=0.79, Fig. 2A, B).

#### Heart rate

Baseline heart rates were stable and similar at 578 ± 11 bpm and 569 ± 8 bpm for AngII and ranitidine+AngII-treated animals, respectively (*P*=0.55, Fig. 2C, D). AngII-infusion significantly but transiently decreased heart rate in ranitidine+AngII (*P*=0.02 vs baseline)—this was borderline significant in AngII-vehicle-treated animals with *P*=0.056 vs baseline (Fig. 2C, D). Heart rates were similar following AngII-infusion as evaluated by Holm-Sidák’s multiple comparisons test (maximum 620 ± 6 vs. 608 ± 10 bpm, *P*=0.45), but AUCs were smaller for ranitidine-treated animals (daytime 11,801 ± 108 vs 11431 ± 161 AU, *P*=0.06, nighttime 12,996 ± 90 vs 12453 ± 148 AU, *P*=0.031, Fig. 2C, D). Heart weight, normalized to body weight (BW) was not affected by ranitidine, averaging 0.59 ± 0.02% of BW (*n*=18) in AngII and 0.59 ± 0.01% of BW (*n*=15) in ranitidine+AngII-treated animals (*P*=0.92).

#### Plasma aldosterone concentration

**Fig. 3:**
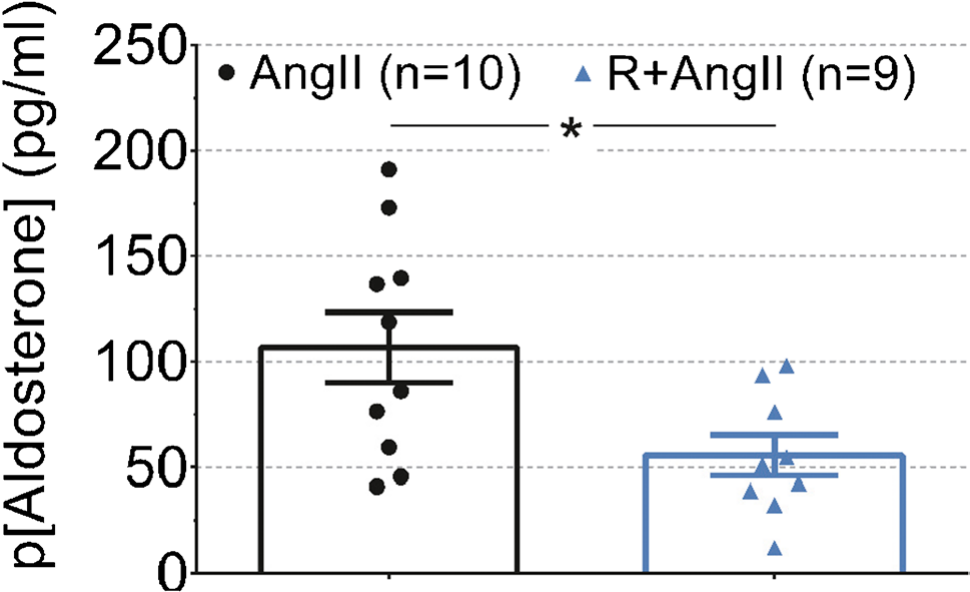
The panel shows plasma aldosterone concentration in resting unstressed C57Bl/6 mice after 24 days of angiotensin II (AngII) and ranitidine+angiotensin II (ranitidine+AngII) treatment. During angiotensin II infusion, plasma levels of aldosterone were reduced significantly by 50% in ranitidine-treated animals, as compared to the *Student’s t*-test for unpaired observations (*P*=0.02). The normality of residuals was evaluated by frequency distribution and quantum-quantum plot. Data are reported as means ± SEM

In resting non-stressed mice co-infused with AngII and ranitidine, plasma aldosterone concentration was significantly lower by 50% on day 24 of the infusion protocol compared to mice infused with AngII alone for 24 days (*P*=0.02, Fig. 3).

### Effect of AngII-induced hypertension with and without treatment with ranitidine on endothelial function and contraction in ex vivo perfused resistance mesenteric arteries

**Fig. 4:**
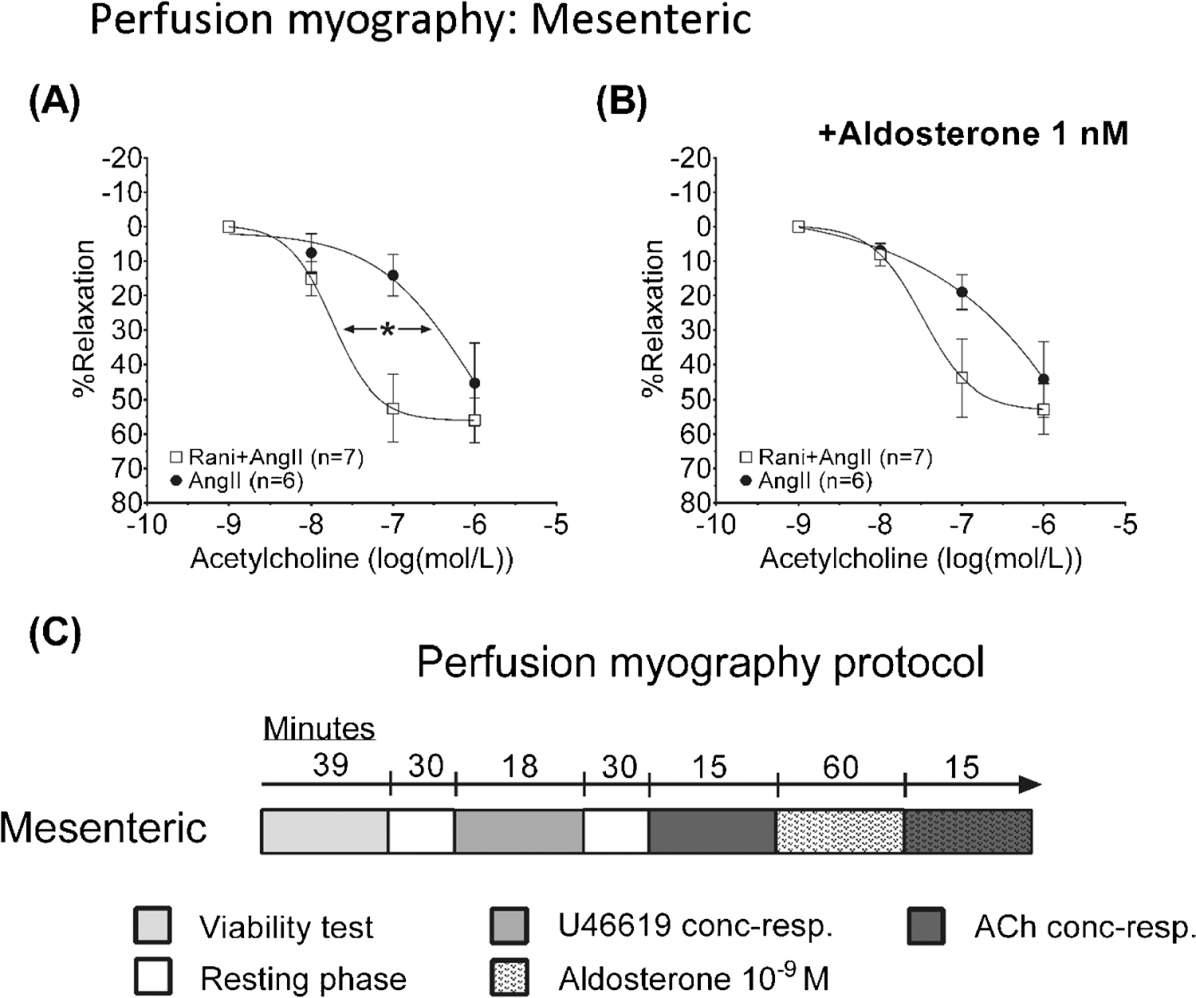
Panels show diameter changes during responses to acetylcholine in isolated perfused mesenteric arteries from C57Bl/6 mice treated in vivo with infusion of angiotensin II (AngII) or combined ranitidine+angiotensin II (ranitidine+AngII). Ex vivo, arteries were pre-constricted with a sub-maximal dose of U46619. The EC_50_ of acetylcholine was significantly lower in arteries from ranitidine+angiotensin II–treated animals as compared with arteries from angiotensin II infused animals ((**A**) EC_50_ −7.6 ± 0.2 vs −6.6 ± 0.3 log(mol/L), *P*=0.03 as compared by *Student’s t-*test). This difference was absent following 1-h incubation with 1 nmol/L aldosterone ((**B**) EC_50_ −7.4 ± 0.3 vs −6.8 ± 0.3 log(mol/L), *P*=0.06 as compared by *Student’s t-*test). There were no differences in maximal responses for any treatment ((**A**) difference 11 ± 9%, *P*=0.6 and (**B**) 8 ± 10%, *P*=0.8 as compared by *Holm-Sidák’s multiple comparisons test*). The protocol for perfusion myography is illustrated in **C**. The normality of residuals was evaluated by frequency distribution and quantum-quantum plot. Data are reported as means ± SEM

The protocol for perfusion myography is illustrated in Figure 4C. Second-order mesenteric arteries were pressurized and allowed to develop a spontaneous tone for 30 min before initiating the protocol.

Arteries were viability-tested to ensure intact contractile and dilatory properties. After washing and resting the arteries, a concentration-response curve to U46619 was generated to establish EC_50_ for stable pre-constriction. After washing and resting, responses to cumulative concentrations of acetylcholine were recorded following which arteries were washed and incubated with 1 nM of aldosterone for 60 min. Responses to cumulative concentrations of acetylcholine were then repeated.

### Baseline characteristics

The average baseline diameters of perfused mesenteric arteries after developing spontaneous myogenic tone for AngII and rani+AngII were 193 ± 3 vs 187 ± 11 µm (*P*=0.67). When challenged with 100 mM KCl, arteries constricted to similar diameters (115 ± 15 vs. 97 ± 10 µm, *P*=0.33). The capacity for endothelium-dependent relaxation was tested in arteries preconstricted by U46619 The agonist (U46619) concentrations required to induce 50% of maximal constriction (EC_50_) between AngII and ranitidine+AngII were 7.4 ± 0.1 and 7.5 ± 0.1 –log[U46619], *P*=0.80, respectively. Acetylcholine (100 µM) evoked dilatation that averaged 69% ± 4 for ranitidine+angiotensin II–treated animals and 64% ± 5 (*P*=0.46) for angiotensin II–treated animals (data not shown graphically).

#### Response to acetylcholine

To evaluate endothelial responsiveness*,* dilatation to cumulatively increasing concentrations of acetylcholine (10^−9^ mol L^−1^ to 10^−6^ mol L^−1^) was determined in perfused arteries sub-maximally constricted with U46619 (10^−7^ mol L^−1^, Fig. 4A). Such dilatations were investigated without pre-treatments and after incubation with extraluminal aldosterone (10^−9^ mol L^−1^, 60 min) [51, 4]. Responses to acetylcholine differed with regard to EC_50_ which was reduced by *in vivo* ranitidine treatment (−7.6 ± 0.2 vs -6.6 ± 0.3 log(mol/L), *P*=0.03, Fig. 4A). This effect of *in vivo* ranitidine was attenuated following incubation for 1 h ex vivo with aldosterone (10^−9^ mol L^−1^, -7.4 ± 0.3 vs -6.8 ± 0.3 log(mol/L), *P*=0.06, Fig. 4B).

### Effect of AngII-induced hypertension with and without treatment with ranitidine on ex vivo isometric force development in conductance segment—aortic rings

**Fig. 5:**
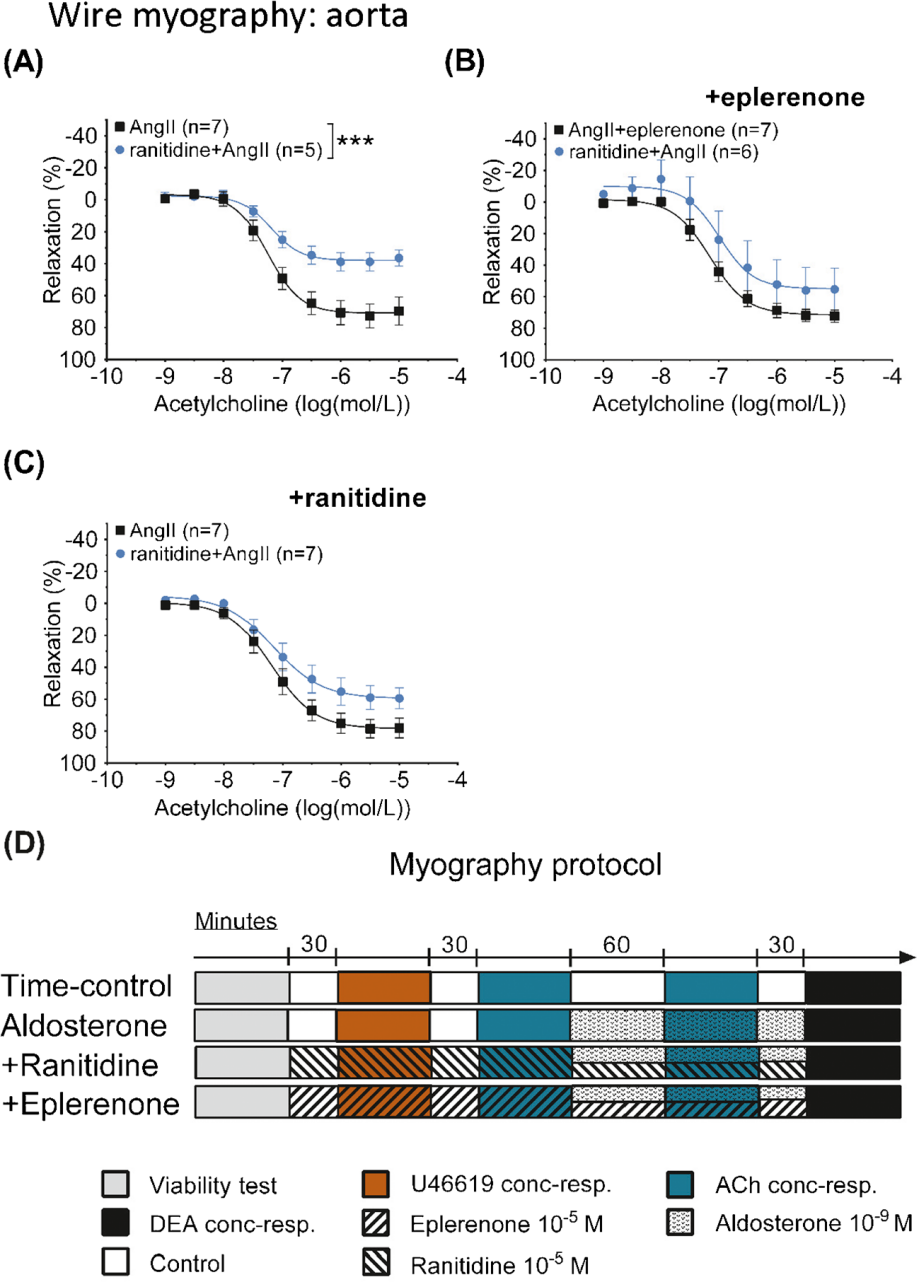
Panels show changes in isometric tension in aortic ring segments isolated from C57Bl/6 mice treated in vivo with angiotensin II (AngII) and combined ranitidine+angiotensin II (ranitidine+AngII). Segments were prestimulated to obtain baseline tension with the thromboxane receptor agonist U46619. Responses to cumulatively increasing concentrations of acetylcholine were tested in parallel in rings that were subjected to ex vivo vehicle, ranitidine and eplerenone. Acetylcholine-induced relaxation was significantly impaired in aortic segments from mice treated *in vivo* with ranitidine as measured by E_max_ ((**A**) 40 ± 4 vs 74 ± 6%, *P*=0.03, as compared by *F-test*) but was similar to segments from AngII-treated mice after *in vitro* 30-min treatment with either mineralocorticoid receptor inhibition by 10 µmol/L eplerenone (65 ± 13 vs 73 ± 5%, *P*=0.24 (**B**)) or H_2_-receptor inhibition by 10 µmol/L ranitidine ((**C**), 64 ± 8 vs 79 ± 7%, *P*=0.08, as compared by *F-test*). An illustration of the myograph protocol is given in **D**. Four segments from four mice were run in parallel with initially three, later four different conditions: time control, then aldosterone treatment (1 nmol/L, initially also time-control conditions), +ranitidine (10 µmol/L) and +eplerenone (10 µmol/L). Segments rested at least 30 min between interventions. Segments were initially viability tested (see details in the “Materials and methods” section). Then a full concentration-response relationship to U46619 (1 nmol/L to 10 µmol/L) was established. After resting, arteries were pre-contracted with EC_50_ for U46619, and a concentration-response relationship to acetylcholine (1 nmol/L to 10 µmol/L) was established. The time control and aldosterone-treated segments were tested under the same conditions as before the aldosterone incubation (upper two protocol, data shown in Fig. 6). Results from those segments before aldosterone incubation were averaged and included as *n*=1. The normal distribution of residuals was evaluated by frequency distribution and quantum-quantum plot. Data are reported as means ± SEM

The myography protocol is given in Figure 5D. Four aortic segments ran in parallel with four different conditions: time control, aldosterone treatment (1 nmol/L), +ranitidine (10 µmol/L), and +eplerenone (10 µmol/L). Segments rested at least 30 min between interventions. Segments were tested initially for viability (see details in the “Materials and methods” section). Then a full concentration response (1 nmol/L–10 µmol/L) to U46619 was established. After resting, arteries were pre-stimulated with U46619 at EC_50_, and a concentration-response to acetylcholine (1 nmol/L to 10 µmol/L) was established. This was repeated after 1 h of aldosterone incubation, and, finally, the NO sensitivity of segments under the four different conditions was tested with cumulative concentrations of the NO-donor diethylamine NONOate (1 nmol/L to 10 µmol/L).

#### Baseline characteristics

Of 72 segments from eighteen animals, 49 segments from 16 animals were included. Normalized internal diameters of aortic segments were 984 ± 15 µm in 26 preparations from nine AngII-treated animals and 969 ± 13 µm in 23 rings from seven ranitidine+AngII-treated animals (*P*=0.45). Challenged with 100 mM KCl, aortic segments developed similar isometric tension up to 2.4 ± 0.2 mN mm^−1^ in AngII and 2.9 ± 0.2 mN mm^−1^ in ranitidine+AngII-treated animals (*P*=0.09). Endothelial viability assessed by relaxation to acetylcholine (10^−5^ mol L^−1^) in U46619 (10^−8^ mol L^−1^)-contracted arteries was 55 ± 4% in AngII and 49 ± 3% in ranitidine+AngII-treated animals (*P* = 0.22). Concentration-tension response curves to U46619 were recorded under control conditions and after pre-treatment of segments for 30 min with either eplerenone or ranitidine. Under those three experimental conditions, the EC_50_ to the thromboxane prostanoid-receptor agonist (which was later used for precontraction) was not significantly different in the aortae of AngII and ranitidine+AngII-treated animals (−8.3 ± 0.1 vs −8.4 ± 0.1, *P*=0.3).

To determine endothelial responsiveness, increasing concentrations of acetylcholine were administered to aorta segments pre-stimulated by EC_50_ of U46619 to 44 ± 3 and 47 ± 4% of E_max_ (*P*=0.65) in AngII and ranitidine+AngII-treated mice, respectively, with no differences between segments. *In vivo* ranitidine treatment significantly reduced the E_max_ in response to acetylcholine ex vivo (74 ± 6% vs 40 ± 4% relaxation, *P*=0.03, Fig. 5A). This difference in reactivity was prevented by 30 min of *in vitro* treatment with either eplerenone or ranitidine (73 ± 5% vs 65 ± 13%, *P*=0.24 and 79 ± 7% vs 64 ± 8%, *P*=0.08, respectively, Fig. 5B, C).

**Fig. 6:**
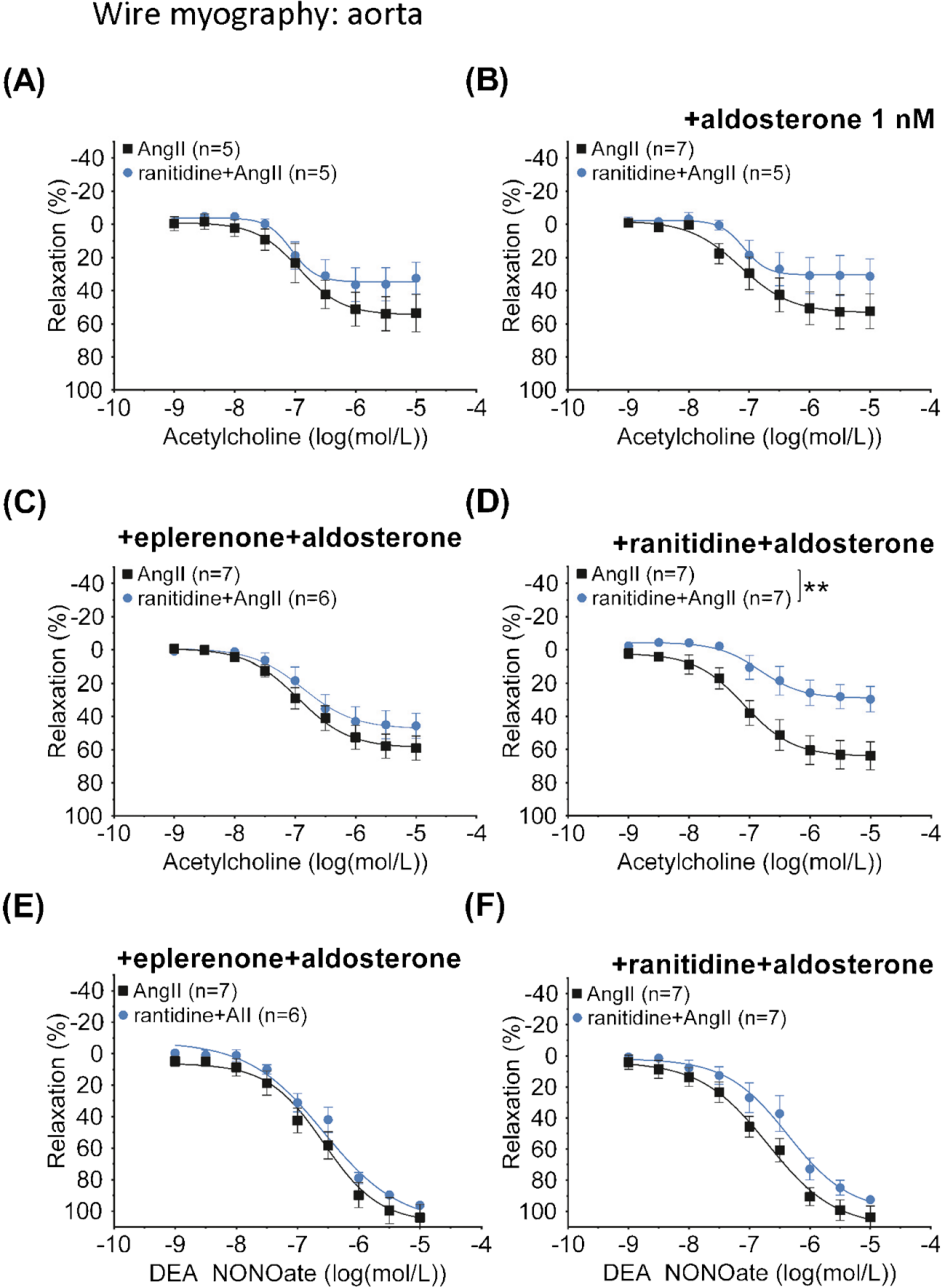
Panels show changes in isometric tension in aortic segments ex vivo isolated from C57Bl/6 mice treated in vivo with angiotensin II (AngII) and ranitidine+angiotensin II (ranitidine+AngII). Baseline tension in all segments was achieved by the thromboxane receptor agonist U46619. Responses to cumulatively increasing concentrations of acetylcholine were tested after a 1-h aldosterone treatment period. Some rings incubated additionally with either eplerenone or ranitidine before exposure to acetylcholine. One-hour aldosterone treatment ex vivo abolished the difference in acetylcholine responses (**B**) as did time (**A**). No effect was detected by combined eplerenone and aldosterone incubation (**C**) but combined ranitidine and aldosterone incubation reintroduced the difference in acetylcholine response ((**D**) 33 ± 6 vs 62 ± 8%, *P*=0.01 as compared by *F-test*) as was seen in Figure 5A. Protocol was terminated by examining relaxation in response to the NO donor diethylamine NONOate. Responses were similar in aortic segments from in vivo angiotensin II and ranitidine+angII-treated animals (data not shown) and were not affected by ex vivo addition of eplerenone (**E**) or ranitidine (**F**). The normality of residuals was evaluated by frequency distribution and quantum-quantum plot. Data are reported as means ± SEM

The difference in acetylcholine-induced relaxation between aortic segments from AngII and ranitidine+AngII-treated animals disappeared with time in the time-control segments (i.e., once removed from the effects *in vivo*) (Fig. 6A). The effect of *in vivo* ranitidine+angII on relaxation was reintroduced by *in vitro* treatment with ranitidine (10^−5^ mol L^−1^) in combination with 1-h administration of aldosterone (10^−9^ mol L^−1^) but only in segments from ranitidine and angiotensin II–treated animals (33 ± 6 vs 62 ± 8%, *P*=0.01, Fig. 6B–D). Increasing concentrations of the NO-donor diethylamine NONOate (DEA NONOate), during U46619 precontraction, induced full and similar relaxations of aortic segments in all experimental groups (not all groups shown, Fig. 6E, F).

## Discussion

The present study was undertaken to examine if the histamine-H_2_-receptor pathway is involved in the hypertensive and vascular effects of aldosterone in the AngII-driven murine hypertension model which promotes the secretion of aldosterone and involves MR activation. The results showed that *in vivo* H_2_-receptor blockade blunted endothelial responsiveness in a conduit artery but improved endothelial responsiveness in resistance arteries which is suggested to involve activation of the MR. H_2_-receptor antagonism did not affect arterial blood pressure, despite significantly reduced plasma-aldosterone level and improved endothelial function in resistance arteries. In aortic segments, ranitidine-induced endothelial dysfunction waned with time but was re-introduced by combined *ex vivo* aldosterone and ranitidine administration. These findings reveal a complex involvement of H_2_ receptors in vascular function during angiotensin II–induced experimental hypertension. The ranitidine-induced reduction of circulating aldosterone concentration on the day of *ex vivo* experiments is a potential confounder in the present experiments, as it may have masked adverse vascular effects of the aldosterone-histamine interaction. The present study confirms a negative chronotropic effect of H_2_ antagonists on heart rate observed previously [25]. Likewise, H_2_ antagonists can acutely but transiently reduce arterial blood pressure [25]. This effect is presumably due to concomitant blockade of α_1_-adrenoceptors [32]. Arterial blood pressure was lower in patients with chronic heart failure that received H_2_ antagonists for 6 months [31]. However, such effects were not observed in mice in the present study; this discrepancy could be due to the variable duration of treatment or to a true species difference. The maintained AngII-mediated hypertension despite significantly lower aldosterone levels indicates that the blood pressure level in AngII-induced hypertension is independent of AngII-mediated increase in circulating aldosterone levels as observed in several previous studies [8, 9, 29, 46]. As is the case for MR antagonists [48], beneficial effects of H_2_ antagonism might not depend only on blood pressure reduction but could also rely on local effects, e.g., improvements of heart morphology [31, 37] and while cardiac fibrosis was not examined in the present study, we found no effect of ranitidine on heart weight which agrees with neutral effect on hypertension. Of note, treatment duration differs between the current study (24 days) whereas in one of the prospective studies, the patients were treated during 6 months [31] while in a cohort with a median follow-up of 11.2 years, consistent use of H_2_ antagonists for more than 1 year confirmed improvements of heart morphology [37].

While most of the classical histamine-dependent effects in allergic reactions are H_1_ receptor–mediated [54], H_2_ receptors are involved in the associated vasodilator responses [28, 50, 54, 55]. Thus, in studies on rat mesenteric arteries and rabbit aortae, histamine-induced dilatations are mediated by H_2_ receptors [28, 34]; likewise, in humans, relaxations to exogenous histamine of isolated uterine arteries are prevented by H_2_ antagonists [55], as are histamine-induced increases in forearm blood flow [50]. Histamine-induced NO release in porcine endothelial cells is mediated by H_2_ receptors [33]. In the present study, *in vivo* ranitidine treatment improved dilatation in mesenteric arteries, while isometric relaxation was significantly impaired in aortic segments as compared to arteries from AngII-treated controls. In the latter, the treatment did not affect relaxations to the exogenous NO donor diethylamine NONOate, demonstrating that the impairment of acetylcholine-induced relaxation is at the level of the endothelial cells. Indeed, a major difference between those two murine blood vessels is that in the former, resistance artery relaxations to acetylcholine are mediated mainly by endothelium-dependent hyperpolarization, while conduit arteries such as the aorta rely on activation of endothelial nitric oxide synthase with production of NO [15, 53, 58]. Thus, the absence of the effect of chronic exposure to ranitidine in mesenteric arteries, which as resistance vessels contribute to the regulation of peripheral resistance, is in line with the absence of the effect of this treatment on arterial blood pressure. H_2_ receptors are involved in contractions evoked by angiotensin II in isolated rabbit aortae [14] and by histamine (at concentrations above 1 µmol L^−1^) in human radial and internal mammary arteries [11]. By contrast, in the present study, H_2_ antagonism did not significantly alter contractions to the thromboxane prostanoid receptor agonist U46619.

As isolated arteries release histamine in response to aldosterone [10], reduced endothelium-dependent relaxation following *in vivo* H_2_ antagonism could be due to either (a) reduced histamine-release dilatation due to the lower circulating level of aldosterone or (b) reduced H_2_-receptor responsiveness in smooth muscle. Relaxations of aortic segments from ranitidine+AngII-treated animals improved to the level of segments of AngII-treated animals during *in vitro* MR antagonism, despite reduced *in vivo* aldosterone levels. The differences in relaxation to acetylcholine in aortic segments, between AngII and ranitidine+AngII-treated animals, also disappeared with prolonged *ex vivo* incubation in a control medium. The reversible nature of the phenomenon suggests that it is mediated by factors present *in vivo*. In line with this interpretation, it was previously shown in murine mesenteric arteries that vascular dysfunction evoked by MR activation is reversed after discontinued exposure to aldosterone [51]. The present study shows that *in vitro* incubation with ranitidine or aldosterone alone does not affect relaxations of aortic segments, irrespective of whether the source of NO is endogenous (endothelium-derived) or exogenous (NO donor). However, the combined *in vitro* incubation with ranitidine and aldosterone re-introduced an endothelial dysfunction to acetylcholine in aortic (but not in mesenteric) segments from ranitidine+AngII-treated animals, but not from AngII-treated animals. This indicates that the interaction of local H_2_ and MR receptors in modulating endothelial release of NO is involved in the response and requires preceding chronic inhibition of the H_2_ receptor, since no acute response is seen *in vitro* in AngII preparations. The finding of a detrimental effect on relaxations to acetylcholine, of combined *in vitro* H_2_-receptor antagonism and MR stimulation contrasts with previous findings, where murine mesenteric arteries *in vitro* co-incubation with the H_2_-receptor antagonist cimetidine and aldosterone prevented aldosterone-induced vascular dysfunction [51]. The present finding could suggest synergistic effects of H_2_ antagonism and MR activation during chronic exposure, e.g., by blocking endogenous activation of H_2_ receptors by histamine derived from vascular cells [10] thereby inhibiting the known increase in cAMP in endothelial cells [23, 33] and by MR-induced eNOS uncoupling and inactivation [44]. This would also explain why no significant reduction of relaxation was detected in mesenteric arteries, since acetylcholine-induced relaxation is mainly NO-dependent in the aorta, whereas it depends on endothelium-derived hyperpolarization (EDH) in mesenteric arteries [15, 53, 58]. Furthermore, as the addition of aldosterone to resistance arteries rendered the difference in acetylcholine-induced dilatation statistically non-significant, one can speculate that the reduced *in vivo* plasma concentrations of aldosterone as induced by H_2_ receptor blockade was beneficial for the endothelial function of resistance arteries as has been previously shown *in vitro* [51].

It was unexpected that plasma aldosterone was lower after ranitidine, but H_2_-receptor dependency of aldosterone release has been observed previously in humans and animals *in vivo* and in adrenals *in vitro* [1, 13, 16, 19, 49]. Thus, the density of mast cells in the adrenal zona glomerulosa correlates with aldosterone secretion in humans [3, 12] with histamine as a likely mediator [12]. This could explain the association between histamine [22, 26], H_2_ receptors [31, 37], and heart failure. The H_2_ receptor is therefore an interesting potential target for intervention.

In conclusion, angiotensin II–induced chronic hypertension in mice is independent of histamine H_2_ receptors. Histamine H_2_ receptors contribute to endothelium- and NO-dependent relaxations in conduit arteries. Histamine by acting on H_2_ receptors supports circulating plasma aldosterone levels and heart rate in AngII-hypertension.

### Perspectives

Histamine and H_2_ receptors may be significant contributors to aldosterone synthesis and release under normal and pathophysiological conditions with elevated circulating levels of angiotensin II. Histamine H_2_-receptor antagonists are widely used drugs with generally benign side effects [59] that could be an attractive and inexpensive alternative to aldosterone synthase inhibitors [7]. 

## Data Availability

Datasets on blood pressure and myography will be available upon reasonable request.
