# Correction to: Histamine H2‑receptor antagonism improves conduit artery endothelial function and reduces plasma aldosterone level without lowering arterial blood pressure in angiotensin II–hypertensive mice

**DOI:** 10.1007/s00424-024-02955-8

**Published:** 2024-03-27

**Authors:** Kasper B. Assersen, Boye L. Jensen, Camilla Enggaard, Paul M. Vanhoutte, Pernille B. L. Hansen

**Affiliations:** 1 Cardiovascular and Renal Research, University of Southern Denmark, J. B. Winsløwsvej 21, Odense C, DK‑5000, Odense, Denmark; 2 State Key Laboratory of Pharmaceutical Biotechnology and Department of Pharmacology and Pharmacy, University of Hong Kong, Hong Kong, China

**Correction to** **:** **Pflügers Archiv - European Journal of Physiology (2024) 476:307–321**

10.1007/s00424-024-02909-0

In the published article, the author Pernille B. L. Hansen was affiliated to 1 and 3. The correct affiliation is only Affiliation 1, University of Southern Denmark. Affiliation 3, AstraZeneca should not be mentioned as no work was performed at or associated with AstraZeneca, thus, affiliation 3 should be removed from the article.

The original article has been corrected.

